# Trends in Incidence and Mortality of Waldenström Macroglobulinemia: A Population-Based Study

**DOI:** 10.3389/fonc.2020.01712

**Published:** 2020-09-10

**Authors:** Xuejiao Yin, Lei Chen, Fengjuan Fan, Han Yan, Yuyang Zhang, Zhenli Huang, Chunyan Sun, Yu Hu

**Affiliations:** ^1^Institute of Hematology, Union Hospital, Tongji Medical College, Huazhong University of Science and Technology, Wuhan, China; ^2^Collaborative Innovation Center of Hematology, Huazhong University of Science and Technology, Wuhan, China

**Keywords:** waldenström macroglobulinemia, surveillance, epidemiology, and end results (SEER), incidence, incidence-based mortality, epidemiology, survival

## Abstract

**Background:** The incidence of Waldenström macroglobulinemia (WM) has increased in certain groups over several decades in the United States. It is unclear whether the increasing incidence is associated with mortality trends.

**Methods:** The incidence and incidence-based mortality (IBM) rates were obtained from the Surveillance, Epidemiology, and End Results (SEER) database (1980–2016) with SEER^*^Stat software. The secular trends stratified by demographic characteristics were analyzed by joinpoint regression.

**Results:** The incidence of WM showed an initial rapid increase from 1980 to 1993 {annual percentage change (APC), 14.1% [95% confidence interval (CI), 10 to 18.4%]}, whereas it began to stabilize from 1993 to 2016 [APC, 0.5% (95% CI, −0.3 to 1.3%)]. The WM IBM trend followed a similar pattern, with a decrease occurring around 1994. The trends in the incidence and mortality significantly differed according to geographic location, race, age, sex, primary site of involvement and subtype, which could help in further investigations into the specific etiology. Moreover, a dramatic increase in the 5-year survival rate from the 1980s to 2010s was observed (47.84 vs. 69.41%).

**Conclusions:** Although both the incidence and IBM of WM continued to increase during the study period, a reduction in the rate of increase occurred around 1993. We believe that further advances in healthcare delivery and research can ensure a low mortality rate. Future studies can use the findings of this paper to monitor the results of WM therapy.

## Introduction

Waldenström macroglobulinemia (WM), a very rare indolent and incurable non-Hodgkin lymphoma (NHL), is characterized by the infiltration of bone marrow and other tissues by lymphoplasmacytic and plasma cells and the presence of circulating immunoglobulin M (IgM) paraproteins in the serum (1). Previous studies based on a large population have shown that the incidence of WM in the United States increased in certain groups from 1988 to 2007 (2). However, it is unclear whether the incidence of this disease had changed over the past 10 years. In addition, it is unknown whether the increased incidence of WM is associated with WM mortality trends. No prior study based on a large population has reported the mortality trend of WM and considered the effects of demographic characteristics on mortality trends.

Castillo et al. (3) suggested that the survival of patients with WM has significantly improved due to the development of novel drugs in recent decades. However, some mixed results have been reported by some studies from Europe (4, 5). If there are indeed notable survival benefits, then they could be detected by a temporal trend analysis of WM incidence and mortality. Nevertheless, to the best of our knowledge, a comparative analysis of these two rates has not been performed in any previous studies.

Therefore, our aim was to explore and compare the trends in incidence and incidence-based mortality (IBM) among patients with WM by age, sex, race, primary site, geographical regions, and subtype using the Surveillance, Epidemiology, and End Results (SEER) cancer registry, including nine registries representing 10% of the US population from 1980 to 2016.

## Materials and Methods

### Data Sources

Incident cases were obtained from the database of incidence–SEER 9 registries of the US National Cancer Institute from 1980 to 2016.

Mortality cases were obtained from the database of IBM–SEER 9 registries of the US National Cancer Institute from 1990 to 2016. IBM cases were different from traditional mortality cases, which linked mortality records to incident cancer cases. The first 10 years (1980–1989) were not included in our IBM analysis to ensure that most of the deaths occurred after 1980 and that IBM rates were not underestimated in the first few years. We chose this interval because the median survival for patients with WM was 6 years from 1991 to 2000 (6).

Survival cases were obtained from the database of incidence–SEER 18 registries of the US National Cancer Institute from 1980 to 2016.

### Study Population Selection

Patients diagnosed with WM as their first malignancy per the International Classification of Diseases for Oncology, third edition, ICD-O-3 histology code 9671/3 [lymphoplasmacytic lymphoma (LPL)] and 9761/3 (WM) were included. The first year of the study period was 1980 because this was the first year in which WM cases were reportable to the SEER database. Patients with WM who were diagnosed only by autopsy or death certificate were excluded. Patients who survived less than a month were excluded from survival analyses since the SEER database recorded their survival time as 0.

Information regarding details of the chemotherapy regimen (e.g., type, dosage, and duration) is limited in SEER. Therefore, the era of diagnosis was divided into the pre-rituximab (1980–2001) and rituximab eras (2002–2016) to explore the effects of novel therapies on survival. Since 2002, 79% of Medicare beneficiaries have received rituximab-based regimens as their initial therapy (7).

### Statistical Analysis

Incidence and mortality rates were calculated with SEER^*^Stat version 8.3.2. All rates were adjusted to the standard population of the US in 2000 and expressed per 100,000 person-years. The joinpoint regression analysis program, version 4.5.0.1, was used to calculate annual percentage changes (APCs), average annual percentage changes (AAPCs), and 95% confidence intervals (CIs) to quantify the incidence and mortality trends according to demographic and tumor characteristics. The program also selected the best-fitting log-linear regression model to ascertain calendar years (i.e., the joinpoints) with significant APCs. All statistical analyses were two-sided, and significance was set at *P* < 0.05. If no cases were reported during a certain year, then this part of the data was excluded when using the Joinpoint software to analyze the incidence and mortality trends.

The Kaplan–Meier method and the log-rank test were used to calculate the overall survival (OS) rates and compare curves. Chi-square tests were used to compare categorical variables, and ANOVA was used to compare continuous variables between populations.

### Ethical Approval

As the SEER database is available to the public, approval from the institutional review board or ethics board was not required.

## Results

### Patient Characteristics in Incidence and Mortality Analysis

A total of 4,472 patients with WM met the inclusion criteria and were included in the incidence analysis; these patients were from nine SEER registries from 1980 to 2016. The patient characteristics are shown in Table 1. Among them, most patients were diagnosed between ages 70 and 79 [1,326 (29.65%)], male [2,598 (58.09%)], white [3,936 (88.01%)], from the Pacific coast region [1,762 (39.4%)], and had a primary site of bone marrow [3,044 (68.07%)]. Of the eligible patients, 2,420 died of WM during the period from 1990 to 2016 and were included in the IBM analysis. Of these deaths, 33.76% occurred in those diagnosed between ages 70 and 79, 58.76% occurred in male patients, and 88.84% occurred in those in the white race group.

**Table 1:** Waldenström macroglobulinemia incidence (1980–2016) and incidence-based mortality (1990–2016): the SEER-9 registry database.

**Characteristic**	**Incidence**		**Incidence-based mortality**	
	**Cases, No. (%)**	**Rate (95% CI)**	**Deaths, No. (%)**	**Rate (95% CI)**
**Overall**	4,472 (100)	0.48 (0.47–0.5)	2,420 (100)	0.34 (0.32–0.35)
**Age, years**				
<50	365 (8.16)	0.06 (0.05–0.06)	136 (5.62)	0.02 (0.02–0.02)
50–59	709 (15.85)	0.65 (0.60–0.70)	265 (10.95)	0.03 (0.03–0.04)
60–69	1,129 (25.25)	1.46 (1.37–1.55)	519 (21.45)	0.07 (0.07–0.08)
70–79	1,326 (29.65)	2.67 (2.53–2.82)	817 (33.76)	0.12 (0.11–0.13)
80+	943 (21.09)	3.20 (3.00–3.41)	683 (28.22)	0.09 (0.09–0.10)
**Sex**				
Male	2,598 (58.09)	0.65 (0.62–0.68)	1,422 (58.76)	0.50 (0.47–0.52)
Female	1,874 (41.91)	0.36 (0.34–0.38)	998 (41.24)	0.23 (0.22–0.24)
**Race^#^**				
White	3,936 (88.01)	0.52 (0.50–0.53)	2,150 (88.84)	0.36 (0.34–0.38)
Black	242 (5.41)	0.29 (0.26–0.33)	142 (5.87)	0.24 (0.20–0.28)
Other^$^	254 (5.68)	0.3 (0.26–0.33)	124 (5.12)	0.18 (0.15–0.22)
**Geographical region**				
East	1,009 (22.56)	0.48 (0.45–0.51)	486 (20.08)	0.30 (0.28–0.33)
Northern plains	1,323 (29.58)	0.49 (0.47–0.52)	778 (32.15)	0.37 (0.35–0.40)
Pacific coast	1,762 (39.4)	0.54 (0.51–0.56)	943 (38.97)	0.37 (0.34–0.39)
Southwest	378 (8.45)	0.31 (0.28–0.35)	213 (8.8)	0.23 (0.20–0.26)
Primary site of involvement^*^				
Bone marrow	3,044 (68.07)	0.33 (0.32–0.34)	1,601 (66.16)	0.22 (0.21–0.23)
Extramedullary disease	1,421 (31.78)	0.15 (0.14–0.16)	815 (33.68)	0.11 (0.11–0.12)
**Subtype recode**				
Lymphoplasmacytic lymphoma	1,878 (41.99)	0.20 (0.19–0.21)	958 (39.59)	0.13 (0.12–0.14)
Waldenström macroglobulinemia	2,594 (58.01)	0.28 (0.27–0.29)	1,462 (60.41)	0.20 (0.19–0.21)

# *The limited number of patients whose race was unknown was excluded from further evaluation in the incidence and incidence-based mortality (IBM) (n = 40 and n = 4, respectively) analyses. Therefore, the percentages of patients of different races in the incidence and IBM analyses do not add up to 100%*.

* *The limited number of patients whose primary site of involvement was unknown was excluded from further evaluation in the incidence and IBM (n = 7 and n = 4, respectively) analyses. Therefore, the percentages of patients with different primary sites of involvement in the incidence and IBM analyses do not add up to 100%*.

$ *The patients whose race was other include American Indian/Alaskan Native and Asian/Pacific Islander*.

*SEER, Surveillance, Epidemiology, and End Results database*.

### Overall Incidence and Mortality Trends

**Figure 1:**
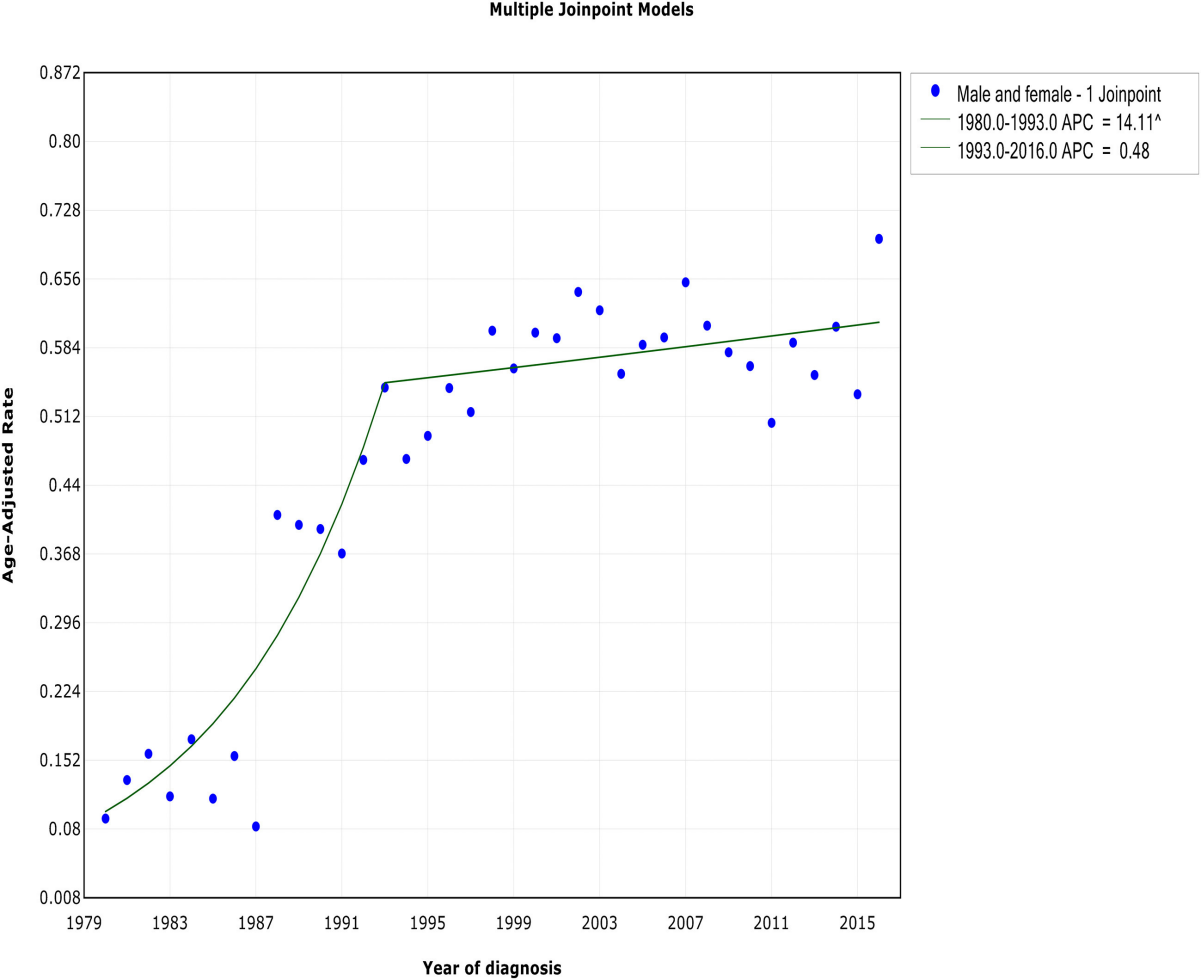
The overall trends in the incidence of Waldenström macroglobulinemia.

**Figure 2:**
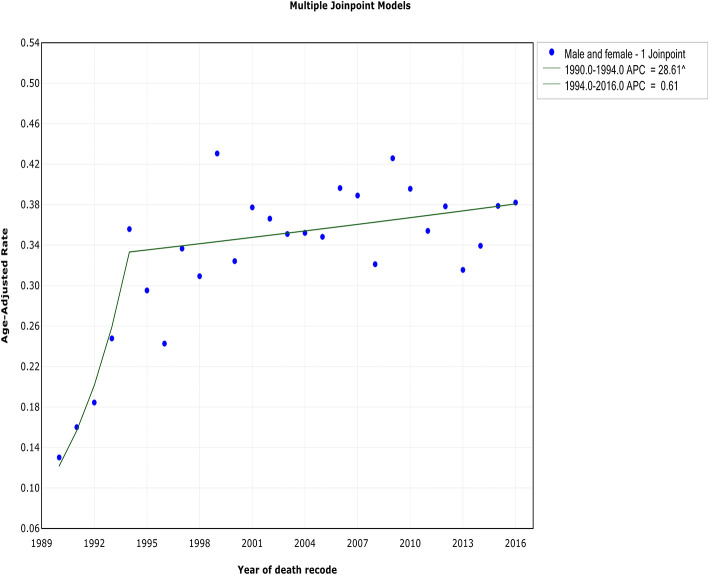
The overall trends in the incidence-based mortality of Waldenström macroglobulinemia.

Although both WM incidence and IBM continued to increase during the study period, a reduction in the rate of increase occurred. This change in the incidence rate occurred ~1 year before the decrease in IBM in 1994. The incidence of WM showed an initial rapid increase from 1980 to 1993 [APC, 14.1% (95% CI, 10 to 18.4%)] and began to stabilize from 1993 to 2016 [APC, 0.5% (95% CI, −0.3 to 1.3%)] (Figure 1, Table 2). In terms of IBM, the rate initially increased at an APC of 28.6% (95% CI, 9.2 to 51.5%) from 1990 to 1994 but slowed from 1994 to 2016 [APC, 0.6% (95% CI, −0.2 to 1.4%)] (Figure 2, Table 3).

**Table 2:** Trends in the incidence rates of Waldenström macroglobulinemia (1980–2016): the SEER-9 registry database.

		**Trend**									
	**Overall (1980–2016)**	**1**		**2**		**3**		**4**		**5**	
	**AAPC (95% CI)**	**Year**	**APC (95% CI)**	**Year**	**APC (95% CI)**	**Year**	**APC (95% CI)**	**Year**	**APC (95% CI)**	**Year**	**APC (95% CI)**
**Overall**	5.2^*^ (3.8 to 6.6)	1980–1993	14.1^*^ (10 to 18.4)	1993–2016	0.5 (−0.3 to 1.3)						
**Age, years**											
<50	0.7 (−1.7 to 3.1)	1980–1997	5.4^*^ (1.1 to 9.9)	1997–2016	−3.4^*^ (−6.1 to −0.7)						
50–59	2.2^*^ (0.8 to 3.7)	1980–2000	7.0^*^ (4.6 to 9.4)	2000–2016	−3.4^*^ (−5.1 to −1.6)						
60–69	4.7^*^ (3.1 to 6.3)	1980–1996	11.6^*^ (8 to 15.3)	1996–2016	−0.5 (−1.7 to 0.7)						
70–79	7.2^*^ (5 to 9.5)	1980–1992	19.3^*^ (12.1 to 27.1)	1992–2016	1.6^*^ (0.7 to 2.6)						
80+	6.5^*^ (3.3 to 9.9)	1980–1990	20.9^*^ (8 to 35.3)	1990–2016	1.5^*^ (0.4 to 2.6)						
**Sex**											
Male	5.4^*^ (3.7 to 7.1)	1980–1993	14.8^*^ (9.9 to 20)	1993–2016	0.4 (−0.5 to 1.3)						
Female	4.9^*^ (3.4 to 6.5)	1980–1993	13.2^*^ (8.9 to 17.7)	1993–2016	0.5 (−0.4 to 1.4)						
**Race**											
White	5.6^*^ (4.1 to 7.2)	1980–1993	14.8^*^ (10.4 to 19.4)	1993–2016	0.7 (−0.1 to 1.6)						
Black	0.4 (−0.9 to 1.7)	1980–2016	0.4 (−0.9 to 1.7)								
Other	0.4 (−0.9 to 1.7)	1980–2016	0.4 (−0.9 to 1.7)								
**Geographical region**											
East	2.4 (−1.4 to 6.4)	1980–1985	−12.5 (−30.1 to 9.5)	1985–1995	13.3^*^ (3.9 to 23.6)	1995–2016	1.3^*^ (0.1 to 2.6)				
Northern plains	5.4^*^ (3.7 to 7.1)	1980–1991	18.1^*^ (12 to 24.5)	1991–2016	0.3 (−0.6 to 1.1)						
Pacific coast	6.5^*^ (3.2 to 9.9)	1980–1989	23.1^*^ (9.4 to 38.4)	1989–2004	4.2^*^ (1.5 to 6.9)	2004–2016	−1.7 (−4.2 to 0.9)				
Southwest	−0.8 (−2.6 to 1.1)	1982–2016	−0.8 (−2.6 to 1.1)								
**Primary site of involvement**											
Bone marrow	17.9^*^ (11.1 to 25.1)	1980–1988	96.5^*^ (49 to 159.2)	1988–2016	1.9^*^ (1.4 to 2.4)						
Extramedullary disease	−0.7 (−5.1 to 4)	1980–1991	−2.1 (−5.9 to 1.8)	1991–1998	11.2^*^ (3.5 to 19.5)	1998–2009	−0.3 (−3 to 2.4)	2009–2012	−25.6 (−54.3 to 21.2)	2012–2016	4.4 (−9.9 to 20.8)
**Subtype**											
Lymphoplasmacytic lymphoma	1.7 (−0.7 to 4.2)	1980–1990	−2.7 (−8.3 to 3.3)	1990–1998	10.9 ^*^(2.6 to 19.9)	1998–2016	0.2 (−1.1 to 1.6)				
Waldenström macroglobulinemia	16.5^*^ (6.8 to 27)	1980–1988	95.9^*^ (30.1 to 194.9)	1988–2016	0.4 (−0.2 to 1)						

* *P < 0.05*.

*APC, annual percentage change; SEER, Surveillance, Epidemiology, and End Results database*.

**Table 3:** Trends in the incidence-based mortality rates of Waldenström Macroglobulinemia (1990–2016): The SEER-9 Registry Database.

		**Trend**					
	**Overall (1990–2016)**	**1**		**2**		**3**	
	**AAPC (95% CI)**	**Year**	**APC (95% CI)**	**Year**	**APC (95% CI)**	**Year**	**APC (95% CI)**
**Overall**	4.5^*^ (1.9 to 7.1)	1990–1994	28.6^*^ (9.2 to 51.5)	1994–2016	0.6 (−0.2 to 1.4)		
**Age, years**							
<50	0.1 (−1.8 to 2.2)	1990–2016	0.1 (−1.8 to 2.2)				
50–59	4.2 (−2.1 to 11)	1990–1993	48.2 (−13.4 to 153.6)	1993–2007	2.7 (−0.8 to 6.3)	2007–2016	−5.1 (−10.2 to 0.1)
60–69	4.1^*^ (0.3 to 8)	1990–1994	25.9 (−1.4 to 60.7)	1994–2016	0.6 (−0.8 to 1.9)		
70–79	4.7^*^ (2.4 to 7.1)	1990–1997	17.8^*^ (8.4 to 28.1)	1997–2016	0.3 (−0.8 to 1.4)		
80+	1.2 (−0.1 to 2.6)	1990–2016	1.2 (−0.1 to 2.6)				
**Sex**							
Male	4.6^*^ (1.2 to 8.2)	1990–1994	30.5^*^ (4.2 to 63.5)	1994–2016	0.5 (−0.5 to 1.6)		
Female	3.5^*^ (1.2 to 5.9)	1990–1997	14.1^*^ (5.1 to 23.9)	1997–2016	−0.2 (−1.4 to 1.1)		
**Race**							
White	5.0^*^ (2.2 to 7.8)	1990–1994	29.2^*^ (8 to 54.5)	1994–2016	1.1^*^ (0.2 to 1.9)		
Black	−0.7 (−2.9 to 1.5)	1990–2016	−0.7 (−2.9 to 1.5)				
Other	−0.8 (−3.2 to 1.6)	1990–2016	−0.8 (−3.2 to 1.6)				
**Geographical region**							
East	4.5^*^ (1.4 to 7.7)	1990–1997	15.2^*^ (3.1 to 28.8)	1997–2016	0.8 (−0.8 to 2.5)		
Northern plains	2.3^*^ (0.3 to 4.4)	1990–1999	8.2^*^ (2.5 to 14.2)	1999–2016	−0.6 (−2.2 to 1)		
Pacific coast	5.0^*^ (0.7 to 9.5)	1990–1994	30 (−2.1 to 72.6)	1994–2016	1 (−0.1 to 2.2)		
Southwest	−1.5 (−3.9 to 0.9)	1991–2016	−1.5 (−3.9 to 0.9)				
**Primary site of involvement**							
Bone marrow	5.3^*^ (2 to 8.8)	1990–1994	35.5^*^ (9.4 to 67.8)	1994–2016	0.6 (−0.3 to 1.6)		
Extramedullary disease	1.3^*^ (0 to 2.7)	1990–2016	1.3^*^ (0 to 2.7)				
**Subtype**							
Lymphoplasmacytic lymphoma	2.4^*^ (1.3 to 3.5)	1990–2016	2.4^*^ (1.3 to 3.5)				
Waldenström macroglobulinemia	4.7^*^ (1.1 to 8.5)	1990–1994	36.7^*^ (8 to 72.8)	1994–2016	−0.2 (−1.3 to 0.9)		

* *P < 0.05*.

*APC, annual percentage change; SEER, Surveillance, Epidemiology, and End Results database*.

### Trends by Sex

**Figure 3:**
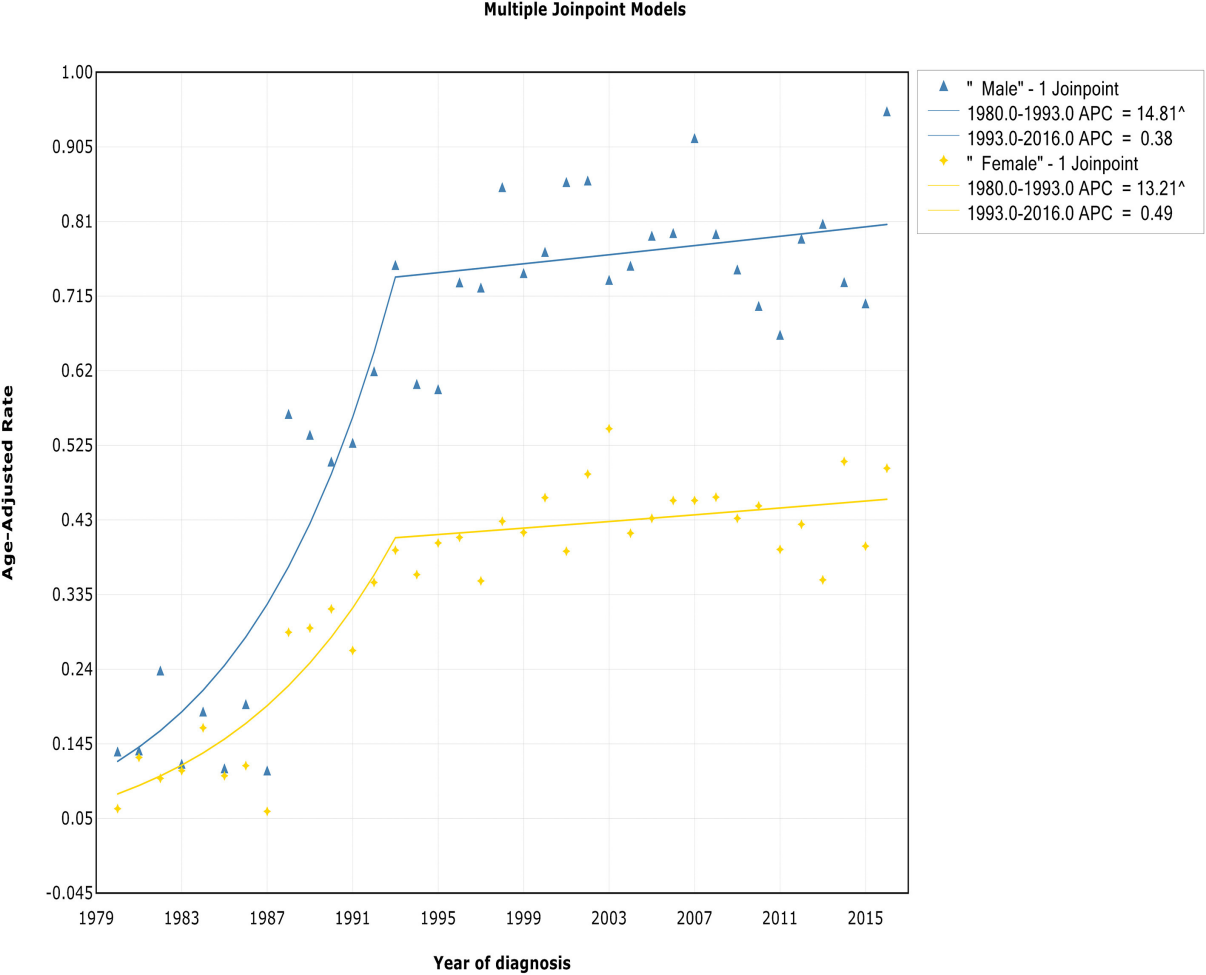
Trends in the annual incidence of Waldenström macroglobulinemia in patients stratified according to sex.

**Figure 4:**
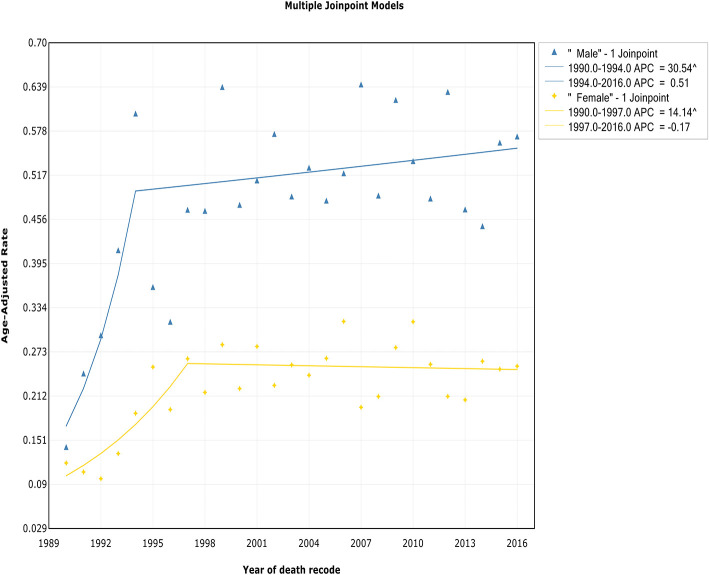
Trends in the annual incidence-based mortality of Waldenström macroglobulinemia in patients stratified according to sex.

The incidence rates in both males and females showed an initial significant increase and then slowed starting in 1993 at rates of 0.4% (95% CI, −0.5 to 1.3%) and 0.5% (95% CI, −0.4 to 1.4%), respectively (Figure 3, Table 2). The IBM rates in males and females followed a similar pattern, with deceleration rates of 0.5% (95% CI, −0.5 to 1.6%) and −0.2% (95% CI, −1.4 to 1.1%) in 1994 and 1997, respectively (Figure 4, Table 3). Although the trends have similar slopes, the annual WM incidence and IBM rates in men are approximately two times and three times, respectively, those in women (i.e., 0.75 vs. 0.39 cases per 100,000 in men vs. women in 1993 for incidence; 0.60 vs. 0.19 cases per 100,000 in men vs. women in 1994 for IBM).

### Trends by Age

**Figure 5:**
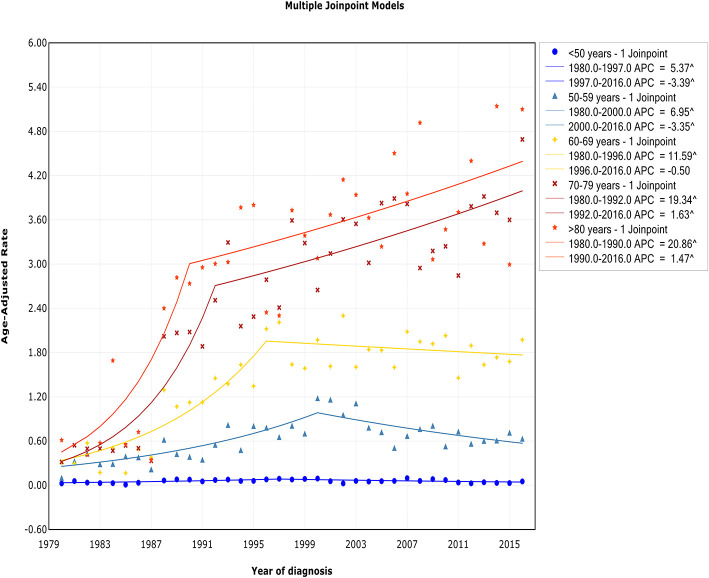
Trends in the annual incidence of Waldenström macroglobulinemia in patients stratified according to age.

The trend of incidence rates among patients diagnosed at ages <50 years, 50–59 years, and 60–69 years exhibited an initial increase and then decreased at APCs of −3.4% (95% CI, −6.1 to −0.7%), −3.4% (95% CI, −5.1 to −1.6%), and −0.5% (95% CI, −1.7 to 0.7%) in 1997, 2000, and 1996, respectively (Figure 5, Table 2). In contrast, for those diagnosed at 70–79 years and 80+ years of age, the incidence rate continued to increase during the study period, but a decline in the rate of increase occurred around 1992 and 1990, respectively. A steep increase in the incidence rate with advancing age was observed. The incidence rate in those aged <50 years was 0.06 of 100,000 per year, but it increased to 0.65 of 100,000 in those aged 50–59 years (11-fold), 1.46 of 100,000 in those aged 60–69 years (24-fold), 2.67 of 100,000 in those aged 70–79 years (45-fold), and 3.20 of 100,000 in those aged >80 years (53-fold).

**Figure 6:**
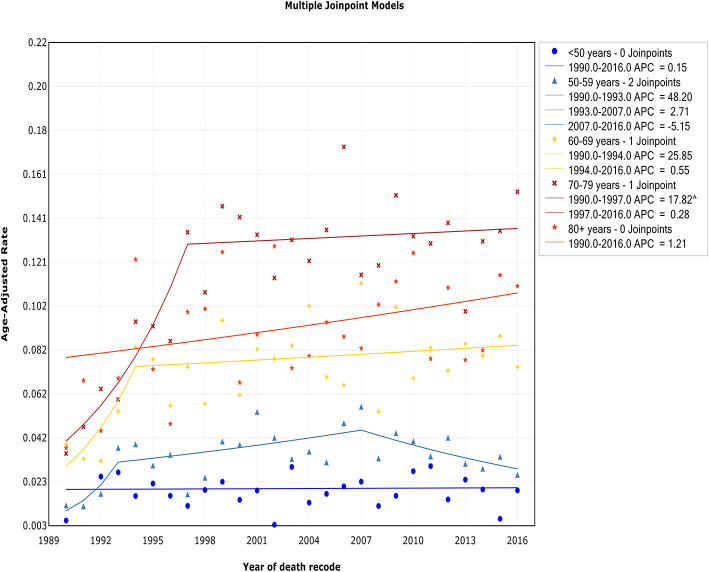
Trends in the annual incidence-based mortality of Waldenström macroglobulinemia in patients stratified according to age.

The trend of IBM rates among patients aged <50 years and >80 years showed a relatively steady level over the study period, and no joinpoint was observed (Figure 6, Table 3). For those aged 50–59 years, 60–69 years, and 70–79 years, the trend of IBM had an initial increase and then stabilized around 2007 [APC, −5.1% (95% CI, −10.2 to 0.1%)], 1994 [APC, 0.6% (95% CI, −0.8 to 1.9%)], and 1997 [APC, 0.3% (95% CI, −0.8 to 1.4%)], respectively.

### Trends by Race

**Figure 7:**
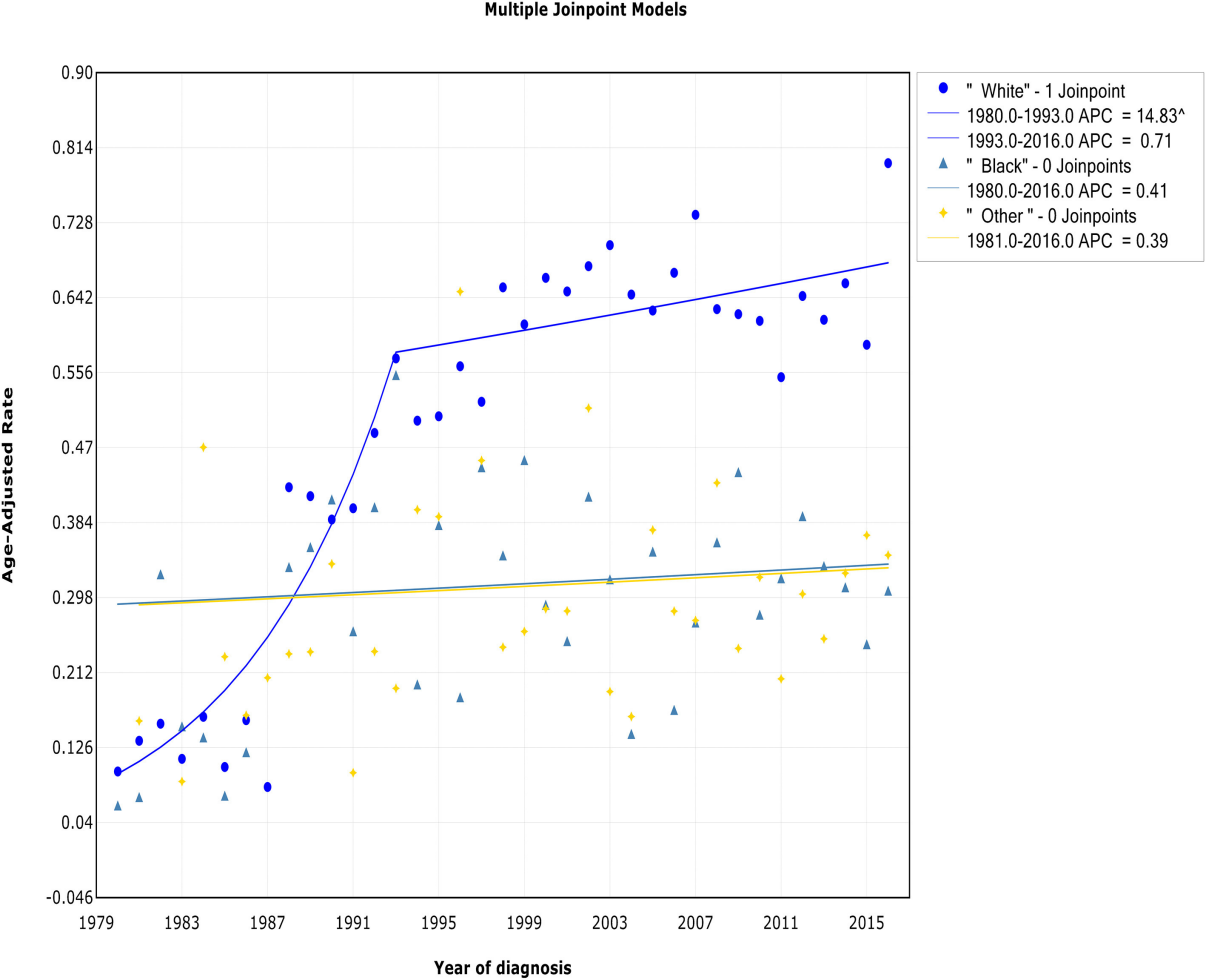
Trends in the annual incidence of Waldenström macroglobulinemia in patients stratified according to race.

**Figure 8:**
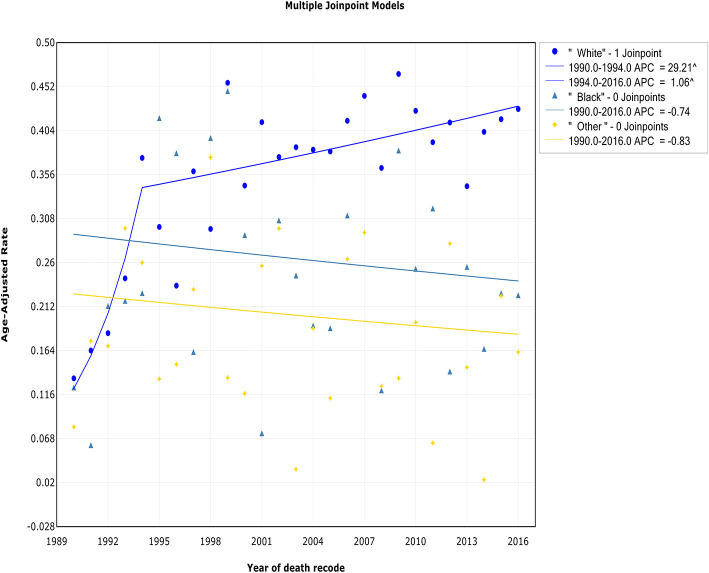
Trends in the annual incidence-based mortality of Waldenström macroglobulinemia in patients stratified according to race.

Both the incidence and IBM trends in the white race group continued to increase over the study period, but a deceleration was observed during 1993 and 1994. The incidence in white patients increased sharply at a rate of 14.8% (95% CI, 10.4 to 19.4%) from 1980 to 1993 and then more slowly from 1993 to 2016 [APC, 0.7% (95% CI, −0.1 to 1.6%)] (Figure 7, Table 2). The trend of the IBM rate in white patients exhibited a similar pattern, with a decrease in the rate of increase from 1994 to 2016 [APC, 1.1% (95% CI, 0.2 to 1.9%)] (Figure 8, Table 3). No changes in the incidence or IBM trends were observed among black patients or other patients over the study period. The annual WM incidence rate was higher among white patients (0.52 of 100,000) than among black patients (0.29 of 100,000) or patients in other racial groups (0.30 of 100,000).

### Trends by Primary Site of Involvement

Both the incidence and IBM rate among patients with bone marrow as the primary site of involvement initially rapidly grew, followed by slowing during the periods 1988–2016 [APC, 1.9% (95% CI, 1.4 to 2.4%)] and 1994–2016 [APC, 0.6% (95% CI, −0.3 to 1.6%)]. When trends in primary extramedullary site-specific incidence rates were analyzed, some joinpoints were observed, but they were not readily interpretable (Supplementary Figure 1, Table 2). This may have been due to mistakes in the data collection program or other anomalies. The trend of IBM rates among patients with extramedullary involvement as the primary site of involvement continued to increase at a rate of 1.3% (95% CI, 0 to 2.7%) during the study period (Supplementary Figure 2, Table 3).

### Trends by Geographical Region

A concomitant initial period of substantial and sustained growth in incidence (Supplementary Figure 3, Table 2) and IBM rates (Supplementary Figure 4, Table 3) was observed for all patients from the eastern and northern plains and Pacific coastal regions, followed by a promising decrease in the rate of increase in the trends in the incidence and mortality rates. There was no joinpoint in the trends in the incidence and IBM for patients from the southwest region.

### Trends by Subtype

For all subtypes, after undergoing a concomitant initial increase in incidence (Supplementary Figure 5, Table 2), all subtypes transitioned to a non-significant increase in the trend of the incidence rate during the same year. For the WM subtype, the incidence increased at an APC of 95.9% (95% CI, 30.1 to 194.9%) during the period from 1980 to 1998 and then exhibited no significant increase thereafter [APC, 0.4% (95% CI, −0.2 to 1%)]. The incidence of the lymphoplasmacytic lymphoma subtype followed a similar pattern, with no significant increase observed from 1998 to 2016 [APC, 0.2% (95% CI, −1.1 to 1.6%)]. In terms of IBM, the IBM rate of patients with the WM subtype continued to increase, showing a deceleration beginning in 1994 (Supplementary Figure 6, Table 3). Therefore, for patients with the WM subtype, the IBM rate was found to decline 4 years earlier than the incidence rate. The trend of IBM among patients with the lymphoplasmacytic lymphoma subtype continued to rise over the study period, with no change observed.

### Survival Analysis

**Figure 9:**
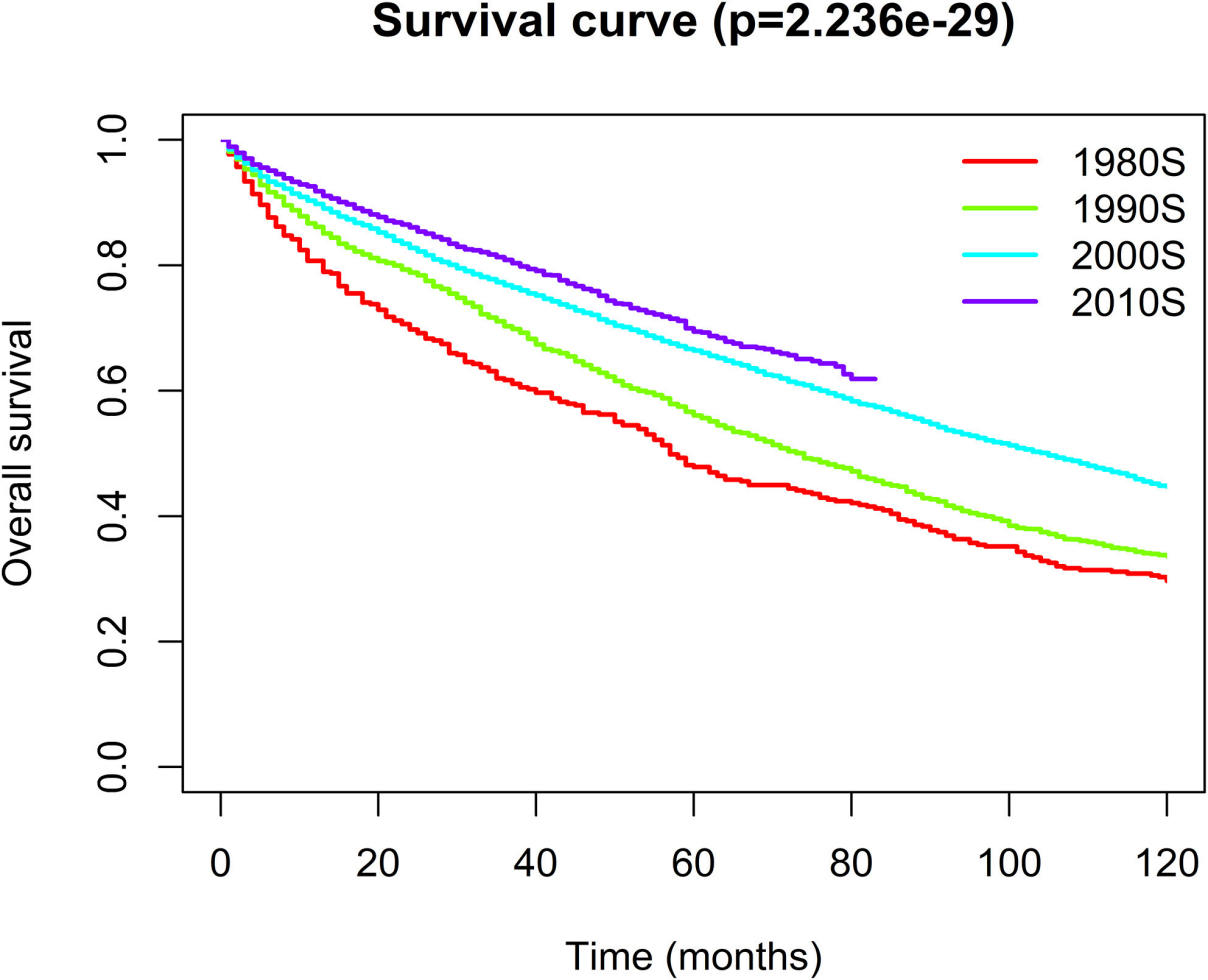
Kaplan–Meier analysis for the overall survival of Waldenström macroglobulinemia. The graph shows increasing survival from the 1980s to 2010s.

Patients with WM have an overall median survival of 96 months, and their 1-, 5-, and 10-year survival rates are 89.4, 64.4, and 42.2%, respectively. The 5-year survival rate significantly increased from the 1980s to the 2010s (47.84 vs. 69.41%) (Figure 9). In addition, survival was compared between the pre-rituximab and rituximab eras to assess the effects of novel therapies on survival. The patients diagnosed in rituximab era had a better OS than patients diagnosed in the pre-rituximab era (Supplementary Figure 7).

## Discussion

To the best of our knowledge, the present study is the first to report comprehensive, up-to-date data on the incidence and mortality of WM based on a large population and to evaluate and systematically compare annual trends in the incidence and mortality of WM according to geographic location, race, age, sex, primary site of involvement, and subtype. We found that both the incidence and IBM of WM continued to increase during the study period, and a reduction in the rate of increase occurred around 1993. This finding may be partly supported by 5-year survival analysis, which indicated that there was a dramatic improvement in the 5-year survival rates from the 1980s to 2010s (47.84 vs. 69.41%).

The present projections of the WM incidence trends may play an important role in assessing the risk factors associated with WM incidence and represent a resource for planning and evaluating WM control programs. We observed that the incidence trend of WM showed an initial rapid increase, whereas it began to stabilize from 1993 to 2016, which was consistent with the findings of previous reports (2, 8). The exact mechanism by which a reduction occurred in the WM incidence rate was unclear; however, several reasons may explain this trend. According to an Italian study, hepatitis C virus (HCV) infection is associated with an increased risk of WM (9). However, researchers have not clearly determined whether the decrease in the WM incidence was related to the decrease in HCV prevalence. Additionally, a randomized controlled trial conducted in the USA reported the opposite conclusion that a relationship does not exist between HCV infection and WM (10). Additional studies are needed to explore the relationship and the exact mechanism underlying the correlation between HCV infection and WM incidence. Genetic predisposition is also related to the development of WM. Castillo et al. (11) showed that there was a 20% increased risk of WM in first-degree family members of patients with WM. In addition, McMaster (12) described some studies focused on investigating family clusters of WM, the longest tracking period of which is nearly six decades, which provides the basis and materials for ongoing genetic research to identify genes that predispose an individual to WM. These investigations may help elucidate the host factors underlying WM development and may be helpful for reducing the WM incidence. Another significant cause may be the misdiagnosis of WM. IgM multiple myeloma (MM) is an infrequent subtype of MM, with an estimated prevalence of 0.5% (13, 14). Due to its rarity and similarity to WM, it is difficult to clinically distinguish these two diseases in most cases. Furthermore, the concentration of IgM varies widely in WM, which makes it impossible to distinguish WM from other lymphoproliferative disorders based on a certain concentration (15). However, with advancements in diagnostic techniques, including laboratory examination, bone marrow aspiration, biopsy evaluation, imaging, and real-time allele-specific polymerase chain reaction (AS-PCR) MYD88 L265P-mutated assay (16), more accurate diagnoses of patients with WM can be achieved (17, 18). Specifically, flow immunophenotyping can better distinguish between lymphoid plasma cells and plasma cells (19, 20). More research is needed to fully understand the etiology of WM.

Mortality rates are a less biased measure to reflect the effectiveness of present treatment modalities and quantify improvements in the management of disease. There was a reduction in the rate of increase in IBM from 1994. In line with our results, a previous study from Sweden that included 1,555 patients with WM showed a reduction in mortality in recent years (5). The potential reasons are listed below. (1) Novel agents with high efficiency and low toxicity, such as rituximab, proteasome inhibitors, immunomodulatory drugs, and B-cell receptor inhibitors, might significantly extend progression-free survival (PFS) (21–23). (2) Notable improvements in supportive care, particularly in the management of infections, cardiovascular disease, and WM-related comorbidities (such as hyperviscosity), have significantly reduced treatment-related mortality. (3) The significant improvement in the diagnostic criteria and evolving classification of WM could better distinguish WM from other B-cell tumors (24). (4) A reduction in age-standardized mortality in the United States could be another cause of the decline in WM mortality (25).

The annual WM incidence and IBM rates in men were approximately two times and three times, respectively, those in women in our analysis, which is consistent with previous studies reporting that male sex is related to an increased risk for many hematological malignancies (26). A previous study from Europe reported incidence rates of WM of 0.73 among males and 0.42 among females per 100,000 persons per year (27). Another study from the United States indicated a higher incidence of WM among males (0.34 of 100,000) than among females (0.17 of 100,000) (28). Although the causal etiological mechanisms are unknown, they may include the factors listed below. (1) Environmental and occupational exposure factors, lifestyle, and health awareness may contribute to the difference (29). (2) A higher response rate to rituximab has been observed in females than in males (30–32). (3) Females produce more vigorous humoral and cellular immune responses than males (33) and have stronger resistance to certain infections, thus reducing infection-related mortality.

We observed a steep increase in the incidence rate with advancing age, which is in line with a multistep carcinogenesis model characterized previously (34). A retrospective study also showed a significantly increased risk of WM with age from 1988 to 2007 (2). The reasons might include the following: (1) Immune senescence and increased risk of chronic inflammation with age may be the main reason for this finding (35, 36). (2) Increased age might mean that when patients exposed to immunotherapy and/or chemotherapy, they might have more protracted stimulation by antigenic agents. A higher affinity for self-antigens may exist in some of the clones selected during homeostatic proliferation and could result in older individuals' autoreactivity (37). (3) With age, the genes involved in malignancies and inflammatory and stress responses (38) are upregulated, while genes associated with lymphoid specificity and function are downregulated (39). (4) With age, the bone marrow structure changes significantly, which contributes to the pathogenesis of many blood diseases (40).

The annual WM incidence rate was higher among white patients (0.52 of 100,000) than among black patients (0.29 of 100,000) and patients in other racial groups (0.30 of 100,000), which is consistent with the findings of previous studies (2). There are significantly different incidence rates of monoclonal gammopathy of unknown significance (MGUS) among different races. IgM MGUS has a lower incidence in black patients than in patients of other races, which might explain the decreased incidence of WM seen in these patients (41).

Geographic region-specific incidence and IBM rates could provide important information on the epidemiological features of cancer. We found that the southwest was the only region where no promising downward trend of incidence and IBM was noted in recent years. One possible cause may be poor quality healthcare delivery systems in this region, leading to delays in the diagnosis, misdiagnosis, and a lack of access to novel therapies. Another possible reason for this phenomenon may be the different cultural beliefs, lifestyle, low-income economic status, health awareness, environmental factors, and occupational exposure factors in this region. In addition, the small number of patients in the area may hinder the discovery of a significant decrease in mortality.

Some limitations based on the information available in the SEER database must be noted. First, the information regarding individual-level factors (e.g., alcohol use, occupation, smoking status, environmental exposure, medical history, and family history) is unavailable in SEER, which may be related to incidence and mortality rates. Second, the information about details of the therapy (e.g., the type, dosage, and duration) is limited. Therefore, we could not assess the impact of treatment on the incidence and mortality trends. Third, some results were based on minor numbers of cases or deaths. Monitoring the incidence and mortality rates of WM over time will provide a better understanding of whether the observed trends persist.

## Conclusions

We found that WM incidence and IBM continued to increase for decades, but a promising downward trend has occurred in recent years. The trend of incidence and mortality differs significantly according to geographic location, race, age, sex, primary site of involvement, and subtype, which may help in further investigations into the specific etiology. Moreover, a dramatic increase in the 5-year survival rate was observed from the 1980s to 2010s (47.84% vs. 69.41%). These findings could provide significant information for understanding the cause of cancer, identifying high-risk populations and developing cancer prevention and intervention strategies to decrease WM-associated morbidity and mortality.

## Data Availability Statement

Publicly available datasets were analyzed in this study. This data can be found here: Surveillance, Epidemiology, and End Results (SEER) database (https://seer.cancer.gov/seerstat/).

## Author Contributions

XY collected and analyzed the data and wrote the paper. LC, FF, HY, YZ, and ZH researched the literature and revised the paper. CS and YH conceived and designed the study, analyzed the data, and wrote the paper. All authors reviewed the paper and approved the final manuscript.

## Conflict of Interest

The authors declare that the research was conducted in the absence of any commercial or financial relationships that could be construed as a potential conflict of interest.

## Supplementary Material

Supplementary Figure 1 Trends in the annual incidence of Waldenström macroglobulinemia in patients stratified according to the primary site of involvement.

Supplementary Figure 2 Trends in the annual incidence-based mortality of Waldenström macroglobulinemia in patients stratified according to the primary site of involvement.

Supplementary Figure 3 Trends in the annual incidence of Waldenström macroglobulinemia in patients stratified according to the geographical region.

Supplementary Figure 4 Trends in the annual incidence-based mortality of Waldenström macroglobulinemia in patients stratified according to the geographical region.

Supplementary Figure 5 Trends in the annual incidence of Waldenström macroglobulinemia in patients stratified according to the subtype recode.

Supplementary Figure 6 Trends in the annual incidence-based mortality of Waldenström macroglobulinemia in patients stratified according to the subtype recode.

Supplementary Figure 7 Kaplan–Meier analysis for Waldenström macroglobulinemia in patients stratified according to the era in which the diagnosis was recorded.

## Abbreviations

WM: Waldenström macroglobulinemia
NHL: non-Hodgkin lymphoma
LPL: lymphoplasmacytic lymphoma
SEER: Surveillance Epidemiology and End Results database
OS: overall survival
CI: confidence interval
APC: annual percentage change
AAPC: average annual percentage change
IBM: incidence-based mortality.
