# Corrigendum: Trends in Incidence and Mortality of Waldenström Macroglobulinemia: A Population-Based Study

**DOI:** 10.3389/fonc.2021.657016

**Published:** 2021-02-18

**Authors:** Xuejiao Yin, Lei Chen, Fengjuan Fan, Han Yan, Yuyang Zhang, Zhenli Huang, Chunyan Sun, Yu Hu

**Affiliations:** ^1^ Institute of Hematology, Union Hospital, Tongji Medical College, Huazhong University of Science and Technology, Wuhan, China; ^2^ Collaborative Innovation Center of Hematology, Huazhong University of Science and Technology, Wuhan, China

**Keywords:** Waldenström macroglobulinemia, surveillance, epidemiology, and end results (SEER), incidence, incidence-based mortality, epidemiology, survival

In the published article, there was an incorrect expression of affiliation 1. Instead of “Tongji Medical College, Institute of Hematology, Union Hospital, Huazhong University of Science and Technology, Wuhan, China”, it should be “Institute of Hematology, Union Hospital, Tongji Medical College, Huazhong University of Science and Technology, Wuhan, China”.

The authors apologize for this error and state that this does not change the scientific conclusions of the article in any way. The original article has been updated.

