# Supplementary figures and images for: Trends in Incidence and Mortality of Waldenström Macroglobulinemia: A Population-Based Study
Source: Front Oncol. 2020 Sep 10;10:1712. doi: 10.3389/fonc.2020.01712 (PMC7511580; doi:10.3389/fonc.2020.01712)

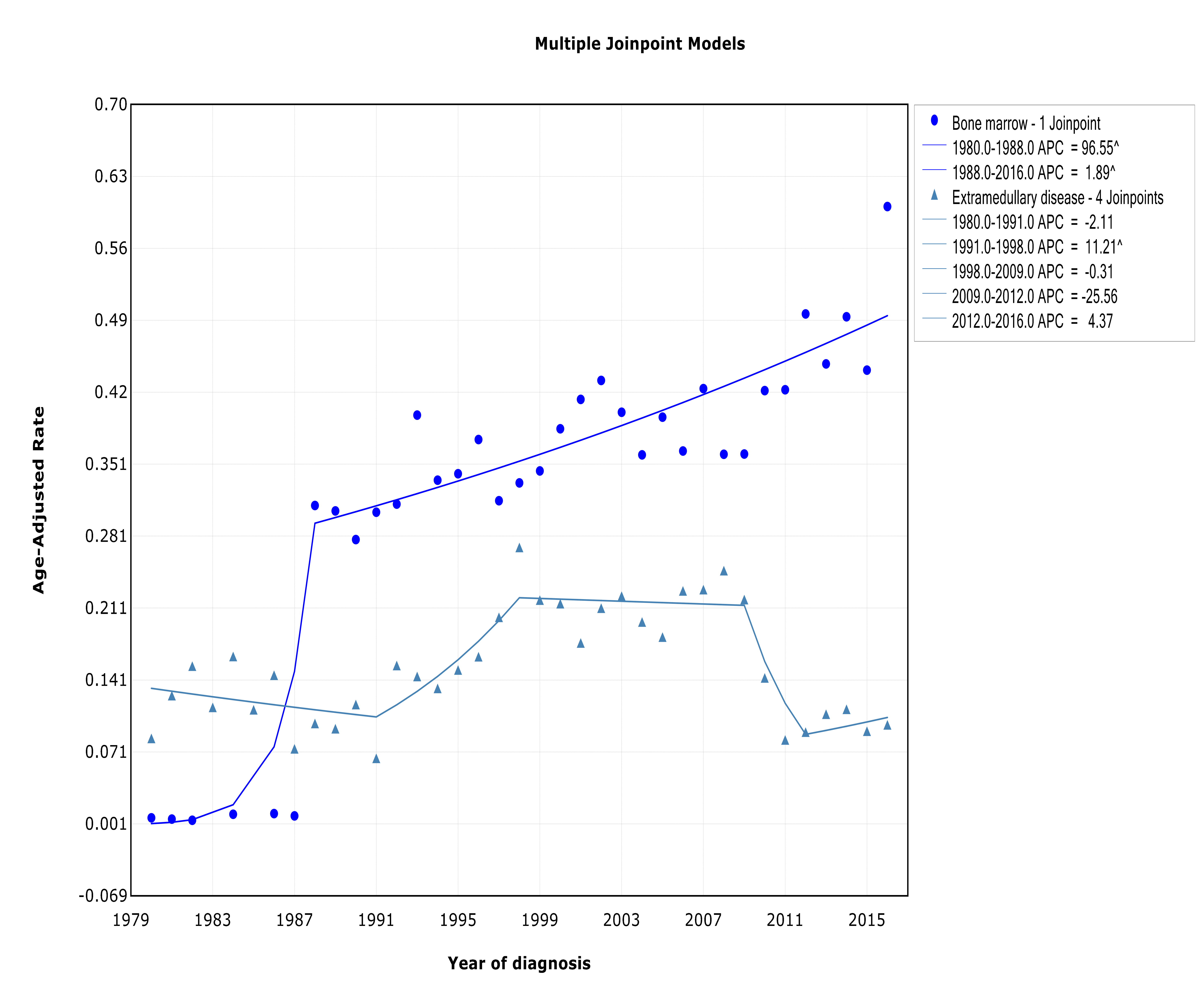

Supplement: Supplementary Figure 1 — Trends in the annual incidence of Waldenström macroglobulinemia in patients stratified according to the primary site of involvement. [file Image_1.TIF]

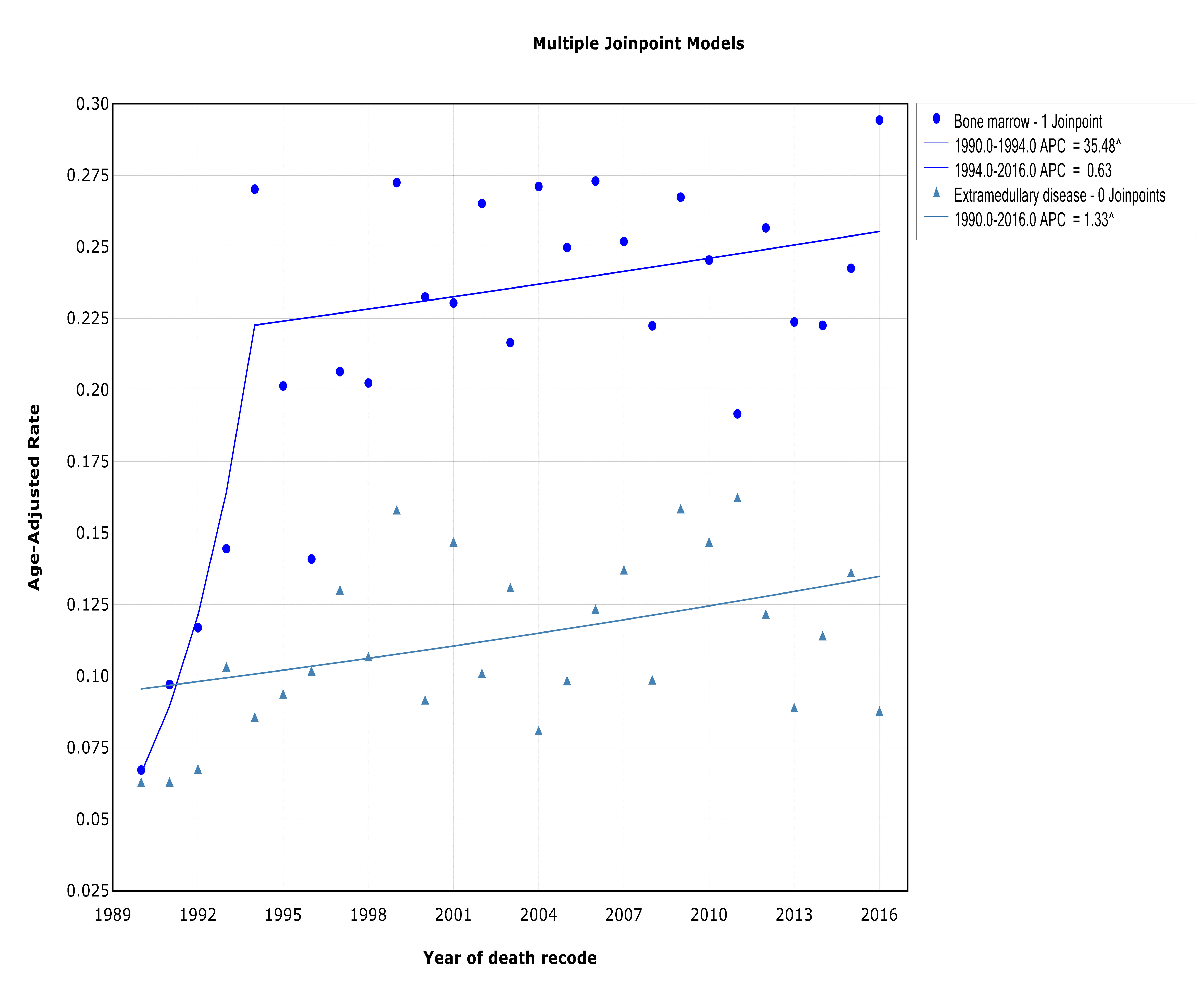

Supplement: Supplementary Figure 2 — Trends in the annual incidence-based mortality of Waldenström macroglobulinemia in patients stratified according to the primary site of involvement. [file Image_2.TIF]

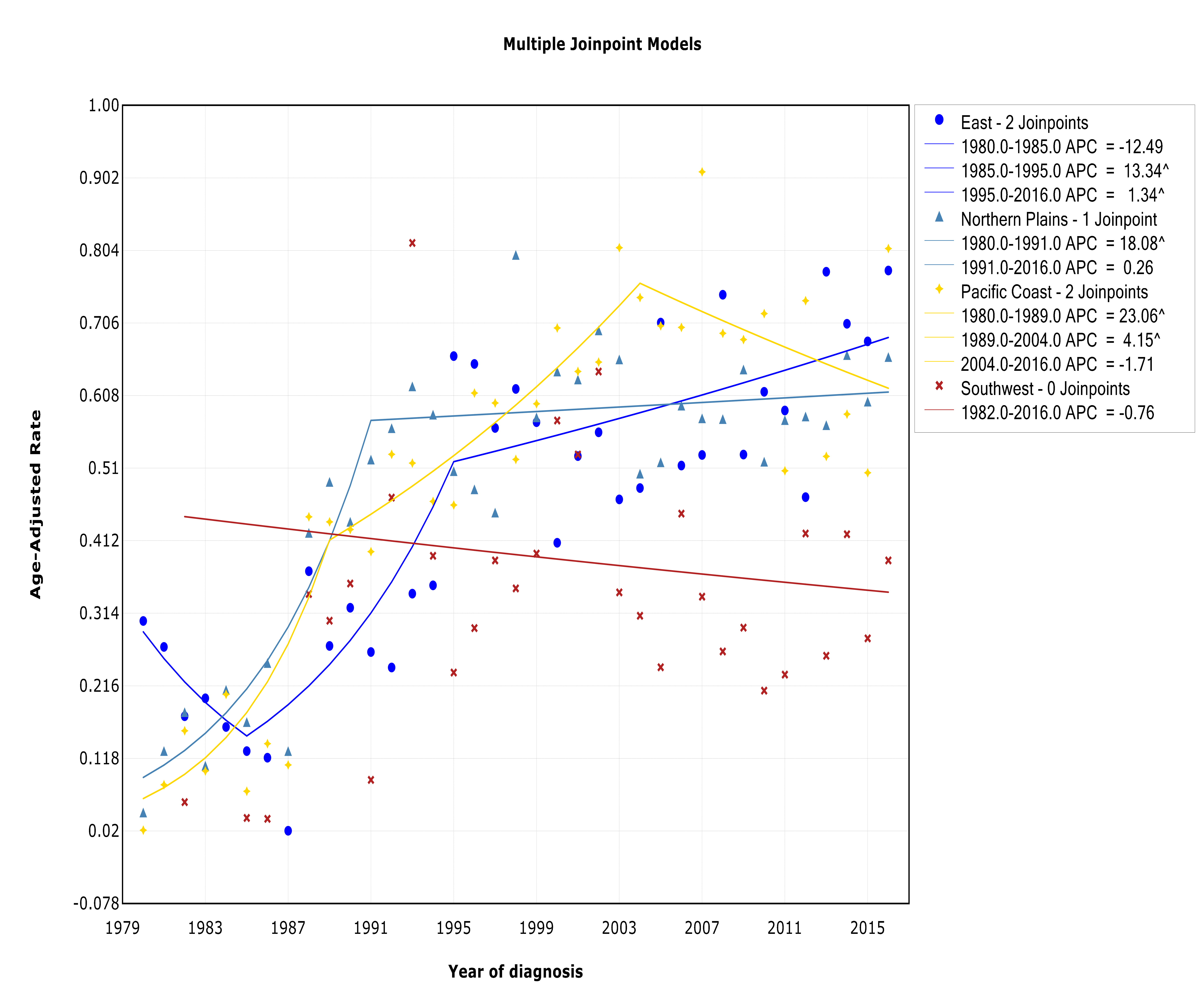

Supplement: Supplementary Figure 3 — Trends in the annual incidence of Waldenström macroglobulinemia in patients stratified according to the geographical region. [file Image_3.TIF]

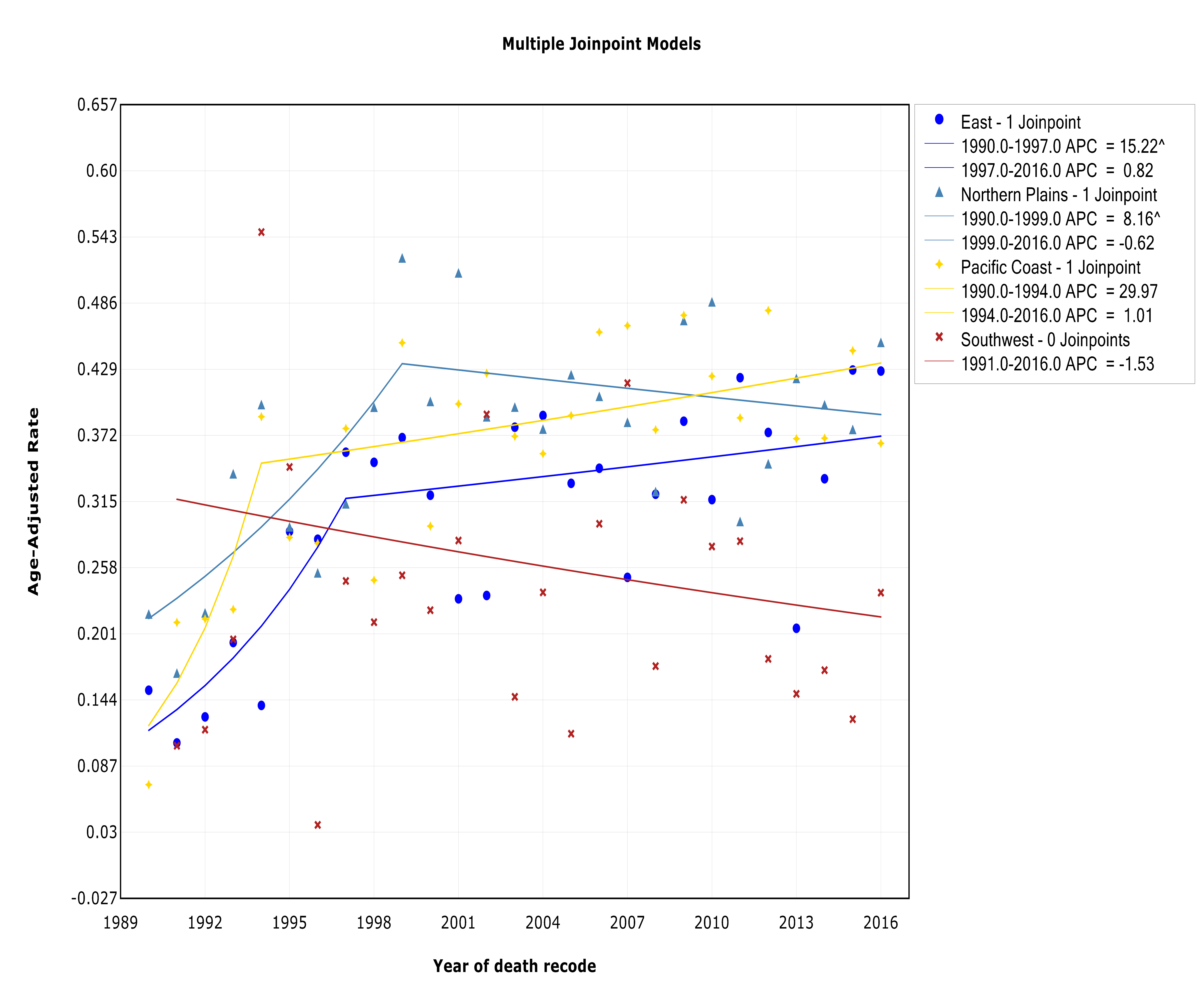

Supplement: Supplementary Figure 4 — Trends in the annual incidence-based mortality of Waldenström macroglobulinemia in patients stratified according to the geographical region. [file Image_4.TIF]

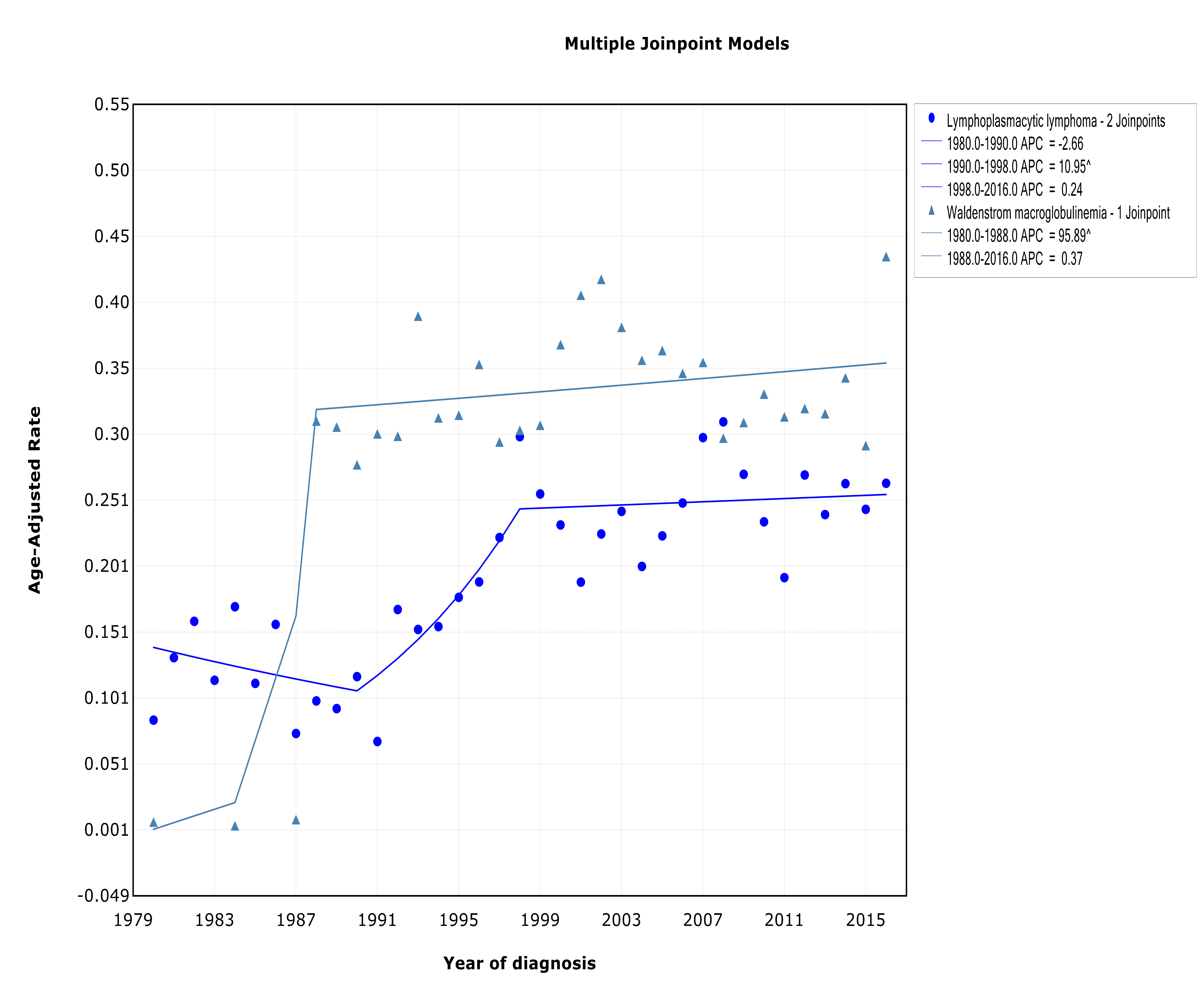

Supplement: Supplementary Figure 5 — Trends in the annual incidence of Waldenström macroglobulinemia in patients stratified according to the subtype recode. [file Image_5.TIF]

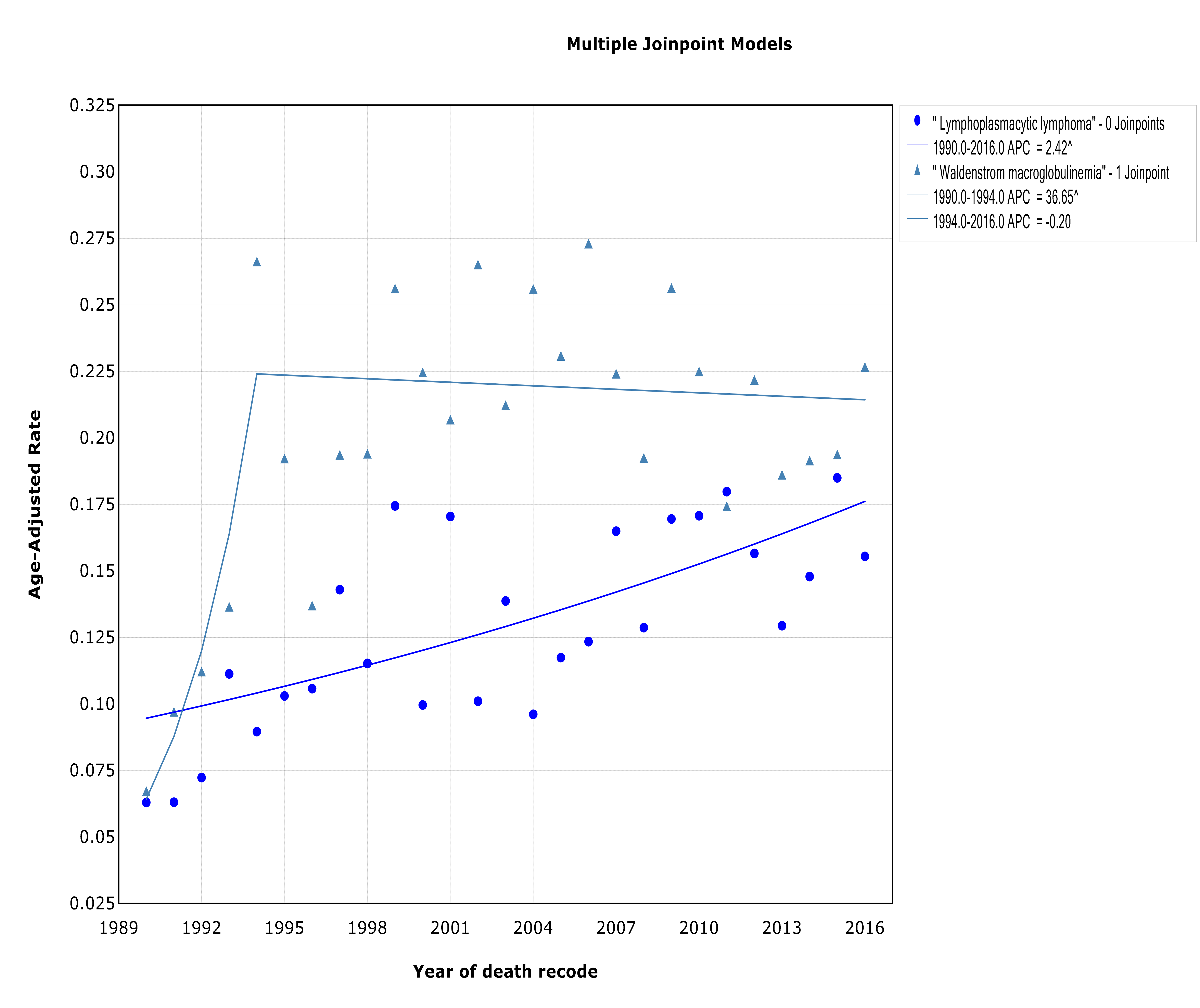

Supplement: Supplementary Figure 6 — Trends in the annual incidence-based mortality of Waldenström macroglobulinemia in patients stratified according to the subtype recode. [file Image_6.TIF]

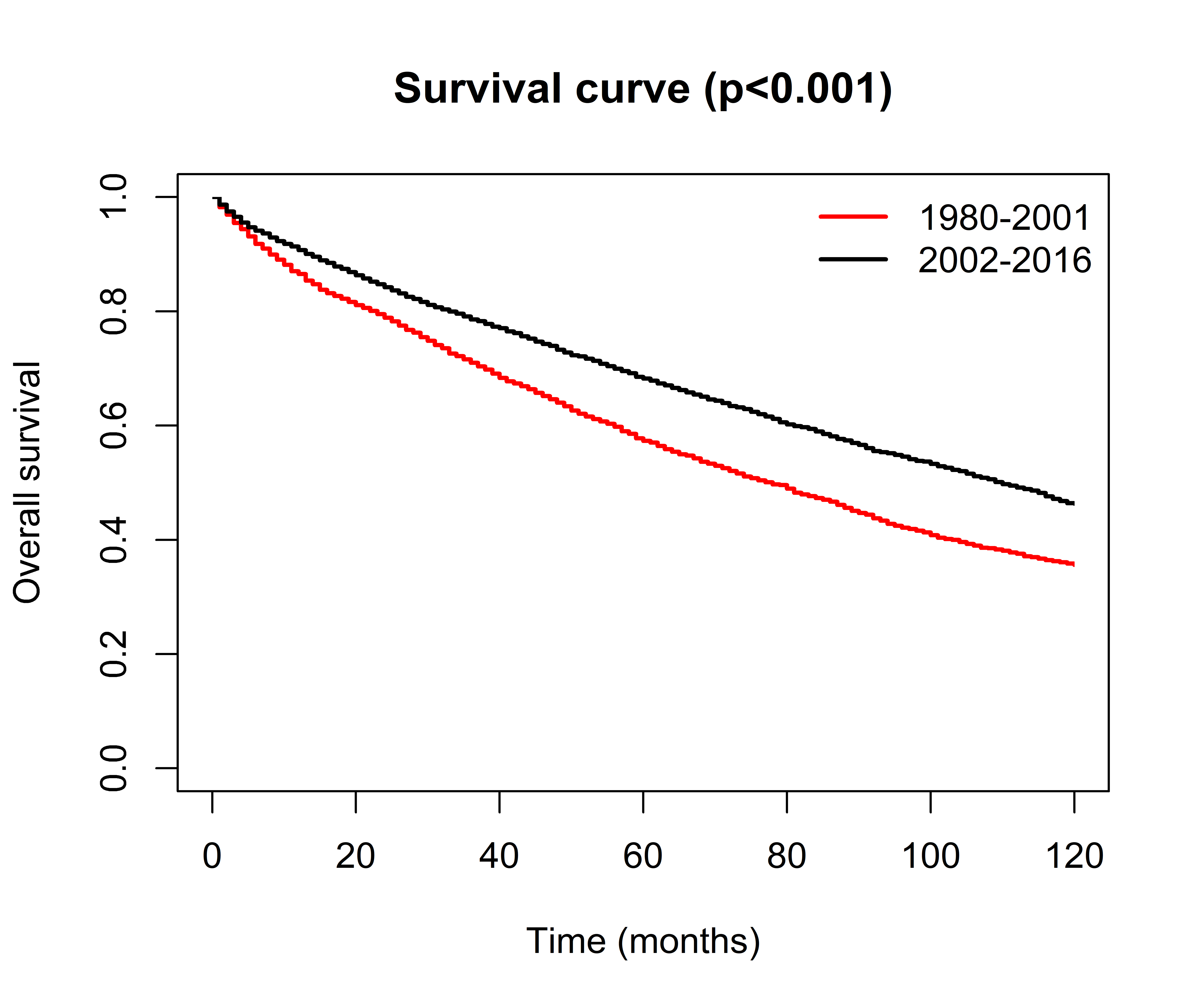

Supplement: Supplementary Figure 7 — Kaplan–Meier analysis for Waldenström macroglobulinemia in patients stratified according to the era in which the diagnosis was recorded. [file Image_7.TIF]
